# The effects of pregnancy intention on the use of antenatal care services: systematic review and meta-analysis

**DOI:** 10.1186/1742-4755-10-50

**Published:** 2013-09-16

**Authors:** Yohannes Dibaba, Mesganaw Fantahun, Michelle J Hindin

**Affiliations:** 1 Department of Population and Family Health, College of Public Health and Medical Sciences, Jimma University, PO Box 378, Jimma, Ethiopia; 2 School of Public Health, College of Health Sciences, Addis Ababa University, PO Box 9086, Addis Ababa, Ethiopia; 3 Johns Hopkins Bloomberg School of Public Health, Johns Hopkins University, 615 N. Wolfe Street, Baltimore, USA

**Keywords:** Unintended pregnancy, Delayed antenatal care, Inadequate antenatal care

## Abstract

**Background:**

There has been considerable debate in the reproductive health literature as to whether unintended pregnancy influences use of maternal health services, particularly antenatal care. Despite the wealth of studies examining the association between pregnancy intention and antenatal care, findings remain mixed and inconclusive. The objective of this study is to systematically review and meta-analyse studies on the association between pregnancy intention and antenatal care.

**Methods:**

We reviewed studies reporting on pregnancy intention and antenatal care from PubMed, Popline, CINHAL and Jstor search engines by developing search strategies. Study quality was assessed for biases in selection, definition of exposure and outcome variables, confounder adjustment, and type of analyses. Adjusted odds ratios, standard errors and sample size were extracted from the included studies and meta-analyzed using STATA version 11. Heterogeneity among studies was assessed using Q test statistic. Effect-size was measured by Odds ratio. Pooled odds ratio for the effects of unintended pregnancy on the use of antenatal care services were calculated using the random effects model.

**Results:**

Our results indicate increased odds of delayed antenatal care use among women with unintended pregnancies (OR 1.42 with 95% CI, 1.27, 1.59) as compared to women with intended pregnancies. Sub-group analysis for developed (1.50 with 95% CI, 1.34, 1.68) and developing (1. 36 with 95% CI, 1.13, 1.65) countries showed significant associations. Moreover, there is an increased odds of inadequate antenatal care use among women with unintended pregnancies as compared to women with intended pregnancies (OR 1.64, 95% CI: 1.47, 1.82). Subgroup analysis for developed (OR, 1.86; 95% CI: 1.62, 2.14) and developing (OR, 1.54; 95% CI: 1.33, 1.77) countries also showed a statistically significant association. However, there were heterogeneities in the studies included in this analysis.

**Conclusion:**

Unintended pregnancy is associated with late initiation and inadequate use of antenatal care services. Hence, women who report an unintended pregnancy should be targeted for antenatal care counseling and services to prevent adverse maternal and perinatal outcomes. Moreover, providing information on the importance of planning and healthy timing of pregnancies, and the means to do so, to all women of reproductive ages is essential.

## Introduction

Maternal health care is important for better maternal, Perinatal and infant health outcomes. High maternal and neonatal mortality rates are associated with inadequate and poor-quality maternal health care, including antenatal care, skilled attendance at birth and postnatal care. Hence, achieving the MDG goal on maternal health requires providing high-quality pregnancy and delivery care, improving sexual and reproductive health care and universal access to all its aspects [1-3]. Indeed, the benefits of healthcare seeking are tremendous particularly in settings where public health resources are limited. Antenatal care is recognized as a key maternal service in improving a wide range of health outcomes for women and children. It provides an opportunity to provide interventions for improving maternal nutrition, to encourage skilled attendance at birth and use of facilities for emergency obstetric care [1,4]. Delayed entry into antenatal care may result in missed opportunities to diagnose pregnancy induced hypertension, gestational diabetes, or sexually transmitted infections.

However, use of these maternal health services is limited, especially in developing countries with high maternal and child mortality. Several individual, household and community level factors have been assessed for the underutilization of maternal health services [5-9]. Among individual factors, studies have considered the role of pregnancy intention in the use of antenatal care. Though, the effects of unintended child bearing remain debated, the committee on unintended pregnancy at the Institute of Medicine concluded that “the consequences of unintended pregnancy are serious, imposing appreciable burdens on children, women, men, and families” [10,11]. Accordingly, a number of studies have assessed the relationship between antenatal care and pregnancy intention finding that women with unintended pregnancies initiate antenatal care late and make inadequate antenatal care visits [12-16]. But, inconsistent findings have been reported in other studies concerning the association between pregnancy intention and antenatal care utilization [17-20]. In particular, the two studies from developing countries (by Marston and Cleland, and Gage) used DHS data of different countries and found an inconsistent association between pregnancy intention and antenatal care. Given the inconsistent findings, and the fact that under-utilization of modern health services are major reasons for poor health in many developing countries of the world, the objective of this study is to systematically review and meta-analyse studies on the association between pregnancy intention and antenatal care.

## Methods

### Search strategies

This systematic review of the literature followed MOOSE (meta-analysis of observational studies in epidemiology) guidelines as proposed by Stroup and colleagues [21]. The data were extracted from already existing published research reports. The literatures used for this review were identified through PubMed, Popline CINHAL and Jstor search engines by developing search strategies. Searches were conducted using terms such as “pregnancy Intention”, “unintended pregnancy”, “unwanted pregnancy”, and “unplanned pregnancy”, “prenatal care”, “antenatal care”, and “maternal health care”. Reference lists of retrieved articles were screened to check whether all pertinent literature was included. Studies that assessed the relationship of pregnancy intention to maternal health, studies that adjusted for confounders and studies published in English were included.

Accordingly, we identified population based cross-sectional studies, cohort studies and case control studies that were reported in English. Reports of data from national or local statistical agencies not reported as published manuscripts were not included. The majority of studies obtained through our search strategies were cross-sectional studies, and few cohort studies were available. We excluded research published before 1980 or data collected earlier than this period.

### Criteria for inclusion of studies

We first identified articles by examining titles, then abstracts for relevance and retrieved the full text of the relevant abstracts for further assessment. The quality of the articles, in terms of internal and external validity, was assessed using a set of criteria developed on the basis of existing instruments for observational studies. Among the criteria used in inclusion of studies were; (1) the author’s provision of explicit definitions for outcomes and exposure variables, (2) whether potential confounders were controlled for in the analysis, (3) studies with data derived from population based sample, and (4) studies with adequate information on the method of ascertainment of pregnancy intention.

Using the above criteria, we extracted proportions, crude and adjusted odds ratios, and their 95% confidence intervals. In cases where odds ratios were not given, we calculated odds ratios and confidence intervals from numerator and denominator data given, but later excluded them due to lack of adjustment for confounders. When beta coefficients and their standard errors were reported, we computed the odds ratio and 95% confidence intervals by taking the inverse natural log of the coefficients.

### Definition of exposure and outcome variables

Comparisons of studies for systematic reviews of this kind are challenged by the variety of ways in which pregnancy intention and antenatal care has been defined. In the majority of the studies included in this analysis, pregnancy intention was assessed using the standard questions used in large surveys such as Demographic and Health Surveys (DHS) and National Survey of Family Growth (NSFG) which asks ‘At the time you became pregnant, did you want to become pregnant then, did you want to wait until later, or did you not want to have any (more) children at all’? In some studies, women were asked whether the pregnancy was planned or not, intended or not or wanted or not wanted. Prospective studies assessed women’s future pregnancy intentions by asking “Are you trying to get (or keep from getting) pregnant now? and how important is avoiding a pregnancy to you?”. Such prospective studies also asked women retrospectively if the pregnancy was intended or not.

Accordingly, intention to become pregnant was classified broadly as intended and unintended, while the latter is further classified into mistimed and unwanted. Intended pregnancy is when the mother indicated that she wanted to become pregnant at that time or sooner. Unintended pregnancy is a pregnancy that had not been wanted at the time conception occurred. Among unintended pregnancies, a distinction is made between unwanted and mistimed pregnancies. Mistimed conceptions are those that were wanted by the woman at some time, but which occurred sooner than they were wanted, and unwanted conceptions are all those that occurred when the woman did not want to have any more pregnancies at all [11].

The main outcomes considered in this review are late initiation of prenatal care and receipt of inadequate (no) prenatal care. Late (delayed) prenatal care was defined as entry in to prenatal care after the first 12 weeks of pregnancy in most of the studies included. Inadequate (no) prenatal care was defined as either less than 4 visits (according to WHO recommendation) or based on the Kessner index to classify whether women received inadequate prenatal care or no prenatal care at all.

### Data analysis

In this analysis, we included studies that adjusted for confounders and that reported odds ratios and their confidence intervals and or standard errors. Thus data were compared including odds ratio (OR) and 95% confidence intervals (CI). STATA software version 11 was used for the analysis. Weighting of the studies is calculated based on the inverse of the variance of the study. Both the fixed and random effects model is reported. But, the random effects model was chosen because it accounts for both random variability and the variability in effects among the studies [22,23]. This means that meta-analysis under random effects assumption recognizes heterogeneity. Forest plots are used to display results graphically. Summary estimates (effect size) with 95% confidence intervals were calculated. Subgroup analyses based on comparison of outcomes for developed and developing countries, and for unintended and intended pregnancies were performed. Heterogeneity was assessed and reported using Cochran’s Q test. Publication bias was checked using Funnel plot.

## Results

### Description of studies

**Figure 1:**
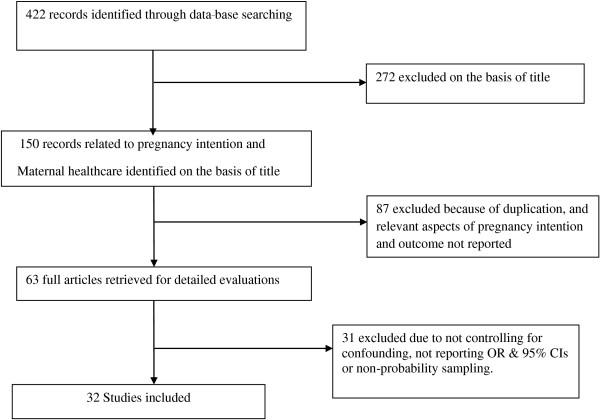
Schematic presentation of studies included in the Analysis.

Figure 1 shows the results of our literature search, study selection and the number of included studies. A total of 422 articles were identified through data base searching of which 272 were excluded on the basis of their title. One hundred fifty (150) articles related to pregnancy intention and maternal health care were identified on the basis of the title but 87 were excluded because of duplication, due to lack of access, and because relevant aspects of pregnancy intention and outcome were not reported. Sixty-three (63) full articles were retrieved for detailed evaluations, of which 31 were excluded because some did not control for confounding, some did not report OR & 95% CIs or applied non probability sampling technique.

In total, 32 observational studies were included in this review (Table 1). Twenty-five of these were cross-sectional studies, 6 were prospective and retrospective cohort studies whereas one was a case control study. More than 252,000 individuals were involved in those studies. Table 1 shows the characteristics of included studies. Several of the studies were secondary analysis of large retrospective cross-sectional surveys such as National Survey of Family Growth (USA studies), and Demographic and Health Surveys (Developing countries). These cross-sectional studies asked pregnancy intention retrospectively after birth, and the duration of the interview varied from few weeks after birth to about five years in surveys such as the DHS. Only 6 were based on data collected prospectively or followed cohorts of pregnant women. These studies measured pregnancy intention before conception or during pregnancy and then re- interviewed women after birth.

**Table 1:** Characteristics of included studies and their assessment of exposure

**No**	**Author**	**Country**	**Design**	**Sample**	**Exposure assessment**	**Response rate**	**Confounders adjusted**
1	Cheng, 2009	USA	CS	9048 women	2 - 9 months postpartum	71%	Maternal age, race/ethnicity, education, marital status, Medicaid status and parity.
2	Eggleston, 2000	Ecuador	CS	3988 women	Women with a pregnancy in the 2 years before the survey interviewed	96.4%	Age, SES, residence, education, number of previous pregnancies
3	Bassani, 2009	Brazil	CS	611 women	Postpartum period	100%	Age, income, education, skin color, parity, satisfaction with pregnancy
4	Magadi, 2000	Kenya	CS	6115 women	Five years prior to the survey	NR	Region/ethnicity, work status, SES, birth order, use of family planning
5	Marston, 2003	5 DHS countries	CS	45,121 women (5 countries)	Five years prior to the survey	NR	Birth order, education, wealth, place of residence
6	Pagnini, 2000	USA	CS	91,585 women	Medical records of women	NR	Race, age, year, psychosocial and behavioral variables
7	Haghpeykar, 2005	USA	CS	300 women	Interviewed during pregnancy	90%	Age, education, income, previous pregnancies, marital status
8	Rodrı’guez, 1997	Spain	CS	409 women	Women admitted for delivery	100%	Social class, education, previous pregnancy, occupation
9	Raghupathy, 1997	Thailand	CS	2754	Women with a birth in the 5 years before the survey	NR	Education, age of mother, income, religion, birth order
10	Braveman,	USA	CS	3071 women	Interviewed during delivery stays in Hospitals	NR	Income, age, education, birth order, race/ethnicity, medical coverage
11	Hulsey, 2000	USA	Historical cohort	1,989 women	Interview as part of cycle V of NSFG	NR	Age, ethnicity, parity, marital status, income, education, employment
12	Marsiglio, 1988	USA	Prospective panel	6,286 women	Interview annually from 1979 - 1988	95.7%	Age, race, residence, education
13	D’Angelo, 2004	USA	CS	25,027 women	Women interviewed for the 1998 PRAMS	NR	Age, marital status, education, race, parity, Medicaid coverage,
14	Waller,	USA	CS	4,898 women	Women and their partners interviewed	83%	Child sex, parental education, parent’s age, parental race/ethnicity, fertility history
15	Biratu, 2000	Ethiopia	CS	1,750 women	Women with a live birth in 12 months before the survey date	100%	Education, age, ethnicity, religion, parity, union type and husband approval
16	Joyce, 2000	USA	CS	4415 women	Late PNC	91%	Child’s sex, mother’s education, region, residence, race/ethnicity
17	Gage, 1998	Kenya & Nambia	CS	6052 & 3877	Women with a birth in the 5 years before the survey	NR	Education, residence, distance to the nearest health facility , ethnicity
18	Hohmann-Marriott	New Zealand	CS	5788	Interview as 1^st^ round of Longitudinal data	NR	Age, education, race/ethnicity, SES, parity and twin status
19	Tariku, 2010	Ethiopia	CS	630 women	Interview during prenatal care	97.1%	Education, parity, means of confirming pregnancy, previous ANC
20	Orr, 2008	USA	CS	913 women	Interview after child birth	NR	Age, education, race/ethnicity, SES, parity
21	Mayor, 1997	USA	Cohort	2032 women	Questionnaire to women who delivered in a facility	70%	Maternal age, education, parity, race, and insurance status
22	Sable , 1998	USA	Case control	2,828 women	3 months postpartum	75%	Maternal age, race, education, Medicaid eligibility, marital status
23	Altfeld, 1998	USA	Cohort	380 women	Interview during pregnancy & Postpartum	99%	Age, race, education, Medicaid, marital status
24	Barrick, 2008	India	Cohort study	3666 women	Interview before conception & after child birth	81.1%	Age, parity, education, asset ownership, autonomy
25	Humbert, 2010	USA	CS	478 women	Interviewed during Postpartum Hospital visit	NR	Age, race, ethnicity, marital status, and parity
26	Weller, 1987	USA	CS/OBS	7,825 women	Women with a live birth in 1980 interviewed	NR	Maternal race residence, and education
27	Behailu, 2009	Ethiopia	CS	620 women	Women who had alive birth in the last year Interviewed	96%	Age, education, residence, ethnicity, marital status
28	Martin, 2007	USA	CS	5404 women & partners	Interview with women and their partners	76.1%	Maternal education, race/ethnicity, marital status, age at birth, household income, employment
29	Potter, 2009	USA	prospective	667 women	Interview with women in prenatal care	NR	Age, race, education, social support and perceived health status
30	Jeffery, 1997	USA	CS	2032	Interview with women coming to delivery	NR	Education, marital status, race, parity
31	Abosie Z., 2009	Ethiopia	CS	691	Women with birth in last 5 years interviewed	97.3%	Parity, number of pregnancies, experience of abortion, still birth , distance from health facility
32	Fenta M., 2005	Ethiopia	CS	642	Women with births in the 12 months before survey	100%	Age, education, ethnicity, marital status, religion, family size

*Cs* = cross-sectional study.

### Pregnancy intention and delayed antenatal care

A total of 19 studies from 9 different countries were included in the analysis for delayed prenatal care. But, the number of studies entered to the software is greater because studies that reported summary measures for unwanted and mistimed pregnancies separately were considered as 2 different studies. In particular, one study conducted in 5 developing countries using DHS data estimated summary measures for each of the 5 countries, and is thus entered as five studies. Sample sizes of the studies ranged from a low of 400 to a high of 90,000. We did sensitivity analysis to exclude studies with the largest and smallest sample size, but that did not change the results significantly. Accordingly, the pooled analysis showed increased odds of delayed prenatal care among women with unintended pregnancies (1.42 with 95% CI, 1.27, 1.59) as compared to women with intended pregnancies. The finding was statistically significant despite the heterogeneity of studies. Sub-group analysis for developed (1.50 with 95% CI, 1.34, 1.68) and developing (1. 36 with 95% CI, 1.13, 1.65) countries showed significant associations. Similarly, sub-group analysis by study design confirmed that in both cohort and cross-sectional studies, there is an increased odds of delayed antenatal care among women with unintended pregnancies compared to women with intended pregnancies (Table 2).

**Table 2:** Stratified and pooled analysis of studies included in meta-analysis of delayed antenatal care and pregnancy intention based on study design and type of country, 1980-2012

**Stratifying variable**	**Sample size**	**Random effects**	**Fixed effects**	**Heterogeneity**	**P**
**Study Design**					
Cross-sectional	121,035	1.43(1.26-1.61)	1.37(1.33-1.41)	281.2	0.001
Prospective cohort	6944	1.36(1.17-1.59)	1.36(1.17-1.59)	5.93	0.762
**Type of country**					
Developed	65,743	1.50(1.34-1.68)	1.64(1.57-1.71)	49.7	0.001
Developing	62,446	1.36(1.13-1.65)	1.26(1.20-1.33)	97.8	0.001
**Pooled estimate**	128,199	1.42(1.27-1.59)	1.37(1.33-1.41)	286.2	0.001

**Figure 2:**
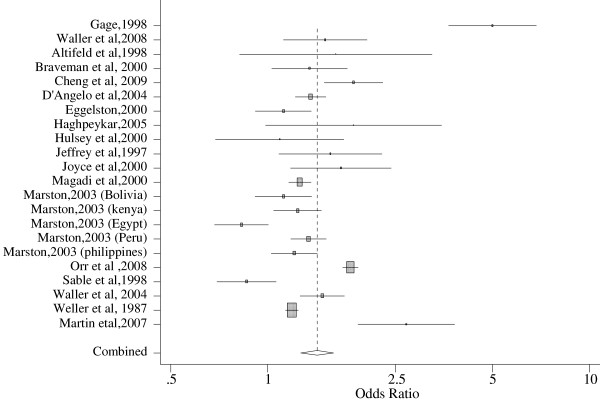
Forest Plot of delayed Antenatal care among women with unintended vs intended pregnancies.

Figure 2 below shows forest plot for delayed antenatal care. The forest plot presents the findings for all studies and the pooled results. An Odds ratio of 1 on the horizontal line helps to interpret the strength of association of the individual studies and the pooled result. Each included study is shown as a horizontal line with a square in the middle, which corresponds to the study’s pooled estimate and 95% confidence interval. The size of the square on the horizontal line shows the study’s weight. Studies with the horizontal line crossing one are the ones that did not show significant associations. At the bottom of the forest plot, the combined effect appears as a diamond whose center shows the average effect size and the extremes show the 95% Confidence Interval (Figure 2).

### Pregnancy intention and inadequate antenatal care

Seventeen (17) studies conducted in 9 different countries were included in the meta analysis for inadequate antenatal care. The result showed significantly higher odds of inadequate antenatal care use among women with unintended pregnancies as compared to women with intended pregnancies (OR 1.64, 95% CI: 1.47, 1.82). There was no heterogeneity problem seen, as shown by the small Q-value of 15.67 and a P-value of 0.096. Moreover, subgroup analysis for developed (OR, 1.86; 95% CI: 1.62, 2.14) and developing (OR, 1.54; 95% CI: 1.33, 1.77) countries showed a statistically significant association. Likewise, sub-group analysis by study design showed increased risk of inadequate antenatal care use among women with unintended pregnancies (Table 3).

**Table 3:** Stratified and pooled analysis of studies included in meta-analysis of inadequate antenatal care and pregnancy intention based on study design and type of country, 1980-2012

**Stratifying variable**	**Sample size**	**Random effects**	**Fixed effects**	**Heterogeneity**	**P**
**Study Design**					
Cross-sectional	48,740	1.66(1.49-1.85)	1.61(1.48-1.75)	17.16	0.192
Prospective cohort	3104	1.56(1.05-2.19)	1.38(1.11-1.71)	4.77	0.092
**Type of country**					
Developed	35,147	1.86(1.62-2.14)	1.86(1.62-2.14)	1.18	0.991
Developing	40,837	1.54(1.33-1.77)	1.50(1.37-1.63)	15.75	0.028
**Pooled estimate**	75,984	1.64(1.47-1.82)	1.58(1.46-1.71)	23.7	0.096

**Figure 3:**
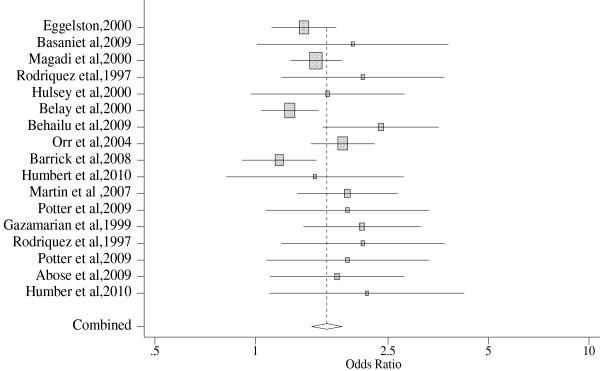
Forest Plot of Inadequate ANC among women with unintended vs intended pregnancies.

Figure 3 shows forest plot for inadequate prenatal care. The forest plot presents the odds ratio and confidence intervals for all studies included and the pooled results. At the bottom of the forest plot, the combined effect appears as a diamond its center showing the average effect size and the extremes show the 95% Confidence Interval.

## Discussion

This study reviewed the evidence on the association between pregnancy intention and antenatal health care, specifically on timely initiation of antenatal care and receipt of adequate antenatal care. Thirty-two observational studies were included into the analysis, selected based on a series of inclusion criteria. We found that there is an increased odds of delayed antenatal care and inadequate antenatal care use among women with unintended pregnancies as compared to women with intended pregnancies. Subgroup analysis done for developing and developed countries also showed a significant association.

However, there were heterogeneities among studies included in the analysis. This was specifically true for analysis on delayed initiation of antenatal care. Although the majority of the studies were cross-sectional studies, there were few cohort/longitudinal studies. Hence, measurement of exposure varied between the studies, although it did not affect the result significantly as shown by the sub-group analysis. They also varied in sample size, but we included studies that controlled for confounders to obtain directly comparable estimates. Even then, the confounders controlled for vary from one study to another. This makes interpretation difficult. Moreover, the bulk of studies on this subject came from the United States, and may not be representative of all developed countries. Publication bias was checked using funnel plot and the result showed that there is no publication bias.

The strength of the systematic review includes; inclusion of studies with adjusted estimates, an extensive literature search, large total sample size of the studied population, a focused research question, and robust effect size and their confidence intervals. There are also some limitations of the study. First, there were few unpublished studies included and majorities were publications on peer reviewed journals and those available online. This is because access to unpublished research reports is difficult. Majority of the studies included are cross-sectional and thus lacks strength to make plausible conclusions. These cross-sectional studies measured exposure sometime after birth and as a result recall bias and ex-post rationalization affect exposure measurement. Previous studies have shown that maternal response to questions of pregnancy intention will vary based on the time lag between the actual pregnancy and the timing of assessment [24-26].

This analysis was restricted to the effects of pregnancy intention on maternal health-care seeking behavior, as measured by timely initiation of ANC and receipt of adequate ANC .This does not mean pregnancy intention is the only factor affecting prenatal care. Several individual, household and community level factors influence the outcomes, and this needs to be kept in mind in interpreting these findings. For instance, among individual level factors, maternal education is consistently and significantly associated with use of antenatal care services. Household socio-economic status, women’s employment, urban residence and parity were among the individual and household level factors associated with the use of antenatal care services in different studies [27-29]. Moreover, community and heath care factors such as women’s autonomy, accessibility, affordability and quality of health services are among the factors reported by different studies as important factors in the utilization of antenatal care services [5,6,30,31].

## Conclusion

The systematic review demonstrated that women’s pregnancy intention influences antenatal care utilization. Sub-group analysis also showed that there are increased odds of delayed and inadequate antenatal care use among women with unintended pregnancies in both developing and developed countries. This has important policy implications, particularly for developing countries with high maternal mortality. Information on the importance of planning and healthy timing of pregnancies should be provided for women of reproductive ages through all appropriate channels. Moreover, maternal health care providers should provide appropriate counseling for women with unintended pregnancies to encourage them to complete the recommended package of antenatal care services.

## Abbreviations

ANC: Antenatal care; CI: Confidence interval; DHS: Demographic and health survey; NSFG: National survey of family growth; WHO: World health organization; OR: Odds ratio.

## Competing interests

The authors declare that they have no competing interests.

## Authors’ contributions

YDW was responsible for the conception, design, collection of studies, analysis and interpretation and the preparation of the draft manuscript. MFA and MJH were involved in the design, collection of studies, the writing, interpretation and revision of the paper. All authors read and approved the final manuscript.

## References

[B1] WHO The World Health Report 2005 - make every mother and child count 2005 Geneva, Switzerland: World Health Organization

[B2] WHO Trends in Maternal Mortality: 1990 to 2008: Estimates Developed by WHO, UNICEF, UNFPA, and the World Bank 2010 Geneva, Switzerland: World Health Organization

[B3] Campbell OM Graham WJ Strategies for reducing maternal mortality: getting on with what works Lancet 2006 368 9543 1284 1299 10.1016/S0140-6736(06)69381-1 17027735

[B4] WHO Antenatal Care in developing Countries: Promises, Achievements and Missed Opportunities. An Analysis of Trends, levels and Differentials, 1990-2001 2003 Geneva, Switzerland: World Health Organization

[B5] Parkhurst JO Penn-Kekana L Blaauw D Balabanova D Danishevski K Rahman SA Health systems factors influencing maternal health services: a four-country comparison Health Policy 2005 73 2 127 138 10.1016/j.healthpol.2004.11.001 15978956

[B6] Stephenson R Baschieri A Clements S Hennink M Madise N Contextual influences on the use of health facilities for childbirth in Africa Am J Public Health 2006 96 1 84 93 10.2105/AJPH.2004.057422 16317204 PMC1470452

[B7] Babalola S Fatusi A Determinants of use of maternal health services in Nigeria–looking beyond individual and household factors BMC Pregnancy Childbirth 2009 9 43 10.1186/1471-2393-9-43 19754941 PMC2754433

[B8] Singh PK Rai RK Alagarajan M Singh L Determinants of maternity care services utilization among married adolescents in rural India PLoS One 2012 7 2 e31666 10.1371/journal.pone.0031666 22355386 PMC3280328

[B9] Celik Y Hotchkiss DR The socio-economic determinants of maternal health care utilization in Turkey Soc Sci Med 2000 50 12 1797 1806 10.1016/S0277-9536(99)00418-9 10798333

[B10] Gipson JD Koenig MA Hindin MJ The effects of unintended pregnancy on infant, child, and parental health: a review of the literature Stud Fam Plann 2008 39 1 18 38 10.1111/j.1728-4465.2008.00148.x 18540521

[B11] Brown S, Eisenberg L The Best Intentions: Unintended Pregnancy and the Well-Being of Children and Families 1995 Washington, DC: National Academies Press 25121228

[B12] Barrick L Koenig MA Pregnancy intention and antenatal care use in two rural north Indian States World Health Popul 2008 10 4 21 37 19550160 PMC4212174

[B13] Bassani DG Surkan PJ Olinto MT Inadequate use of prenatal services among Brazilian women: the role of maternal characteristics Int Perspect Sex Reprod Health 2009 35 1 15 20 10.1363/3501509 19465344

[B14] Eggleston E Unintended pregnancy and women’s use of prenatal care in Ecuador Soc Sci Med 2000 51 7 1011 1018 10.1016/S0277-9536(00)00010-1 11005389

[B15] Hulsey TM Association between early prenatal care and mother’s intention of and desire for the pregnancy J Obstet Gynecol Neonatal Nurs 2001 30 3 275 282 May-Jun 10.1111/j.1552-6909.2001.tb01545.x 11383950

[B16] Orr ST James SA Reiter JP Unintended pregnancy and prenatal behaviors among urban, black women in Baltimore, Maryland: the Baltimore preterm birth study Ann Epidemiol 2008 18 7 545 551 10.1016/j.annepidem.2008.03.005 18504137

[B17] Marston C Cleland J Do unintended pregnancies carried to term lead to adverse outcomes for mother and child? An assessment in five developing countries Popul Stud (Camb) 2003 57 1 77 79 10.1080/0032472032000061749 12745811

[B18] Altfeld S Handler A Burton D Berman L Wantedness of pregnancy and prenatal health behaviors Women Health 1997 26 4 29 43 9525267 10.1300/j013v26n04_03

[B19] Joyce TJ Kaestner R Korenman S The effect of pregnancy intention on child development Demography 2000 37 1 83 94 10.2307/2648098 10748991

[B20] Gage A Premarital Childbearing, Unwanted Fertility and Maternity Care in Kenya and Namibia Popul Stud 1998 52 1 21 34 10.1080/0032472031000150156

[B21] Stroup DF Berlin JA Morton SC Meta-analysis of Observational Studies in Epidemiology, A Proposal for Reporting JAMA 2000 283 2008 2012 10.1001/jama.283.15.2008 10789670

[B22] Olkin I Diagnostic statistical procedures in medical meta-analyses Stat Med 1999 18 17–18 2331 2341 Sep 15-30 10474143 10.1002/(sici)1097-0258(19990915/30)18:17/18<2331::aid-sim259>3.0.co;2-l

[B23] Higgins JP Thompson SG Deeks JJ Altman DG Measuring inconsistency in meta-analyses BMJ 2003 327 7414 557 560 Sep 6 10.1136/bmj.327.7414.557 12958120 PMC192859

[B24] Koenig MA Acharya R Singh S Roy TK Do current measurement approaches underestimate levels of unwanted childbearing? Evidence from rural India Popul Stud (Camb) 2006 60 3 243 256 10.1080/00324720600895819 17060052

[B25] Santelli J Rochat R Hatfield-Timajchy K Gilbert BC Curtis K Cabral R The measurement and meaning of unintended pregnancy Perspect Sex Reprod Health 2003 35 2 94 101 Mar-Apr 10.1363/3509403 12729139

[B26] Tsui AO McDonald-Mosley R Burke AE Family planning and the burden of unintended pregnancies Epidemiol Rev 2010 32 1 152 174 10.1093/epirev/mxq012 20570955 PMC3115338

[B27] Jat RT Ng N Sebastian M Factors affecting the use of maternal health services in Madhya Pradesh state of India: a multilevel analysis Int J Equity Health 2011 10 59 10.1186/1475-9276-10-59 22142036 PMC3283453

[B28] Babalola S Fatusi A Determinants of use of maternal health services in Nigeria – looking beyond individual and household factors BMC Pregnancy Childbirth 2009 2009 9(43) 10.1186/1471-2393-9-43 PMC2754433 19754941

[B29] Tewdros B G/mariam A Dibaba Y Factors affecting antenatal care utilization in Yem Special District, South western Ethiopia Ethiopian Journal of health sciences 2009 19 1 45 51

[B30] Worku AG Yalew AW Afework MF Factors affecting utilization of skilled maternal care in Northwest Ethiopia: a multilevel analysis BMC Int Health Hum Rights 2013 13 20 10.1186/1472-698X-13-20 23587369 PMC3639034

[B31] Basinga P Gertler PJ Binagwaho A Soucat AL Sturdy J Vermeersch CM Effect on maternal and child health services in Rwanda of payment to primary health-care providers for performance: an impact evaluation Lancet 2011 377 9775 1421 1428 10.1016/S0140-6736(11)60177-3 21515164

